# Chronic insomnia, REM sleep instability and emotional dysregulation: A pathway to anxiety and depression?

**DOI:** 10.1111/jsr.14252

**Published:** 2024-05-29

**Authors:** Dieter Riemann, Raphael J. Dressle, Fee Benz, Kai Spiegelhalder, Anna F. Johann, Christoph Nissen, Elisabeth Hertenstein, Chiara Baglioni, Laura Palagini, Lukas Krone, Michael L. Perlis, Katharina Domschke, Mathias Berger, Bernd Feige

**Affiliations:** ^1^ Department of Psychiatry and Psychotherapy, Medical Center‐University of Freiburg, Faculty of Medicine University of Freiburg Freiburg Germany; ^2^ Institute of Medical Psychology and Medical Sociology, Faculty of Medicine University of Freiburg Freiburg Germany; ^3^ Department of Psychiatry, Faculty of Medicine University of Geneva Geneva Switzerland; ^4^ University Hospital of Psychiatry and Psychotherapy University of Bern Bern Switzerland; ^5^ Division of Psychiatric Specialties, Department of Psychiatry Geneva University Hospitals (HUG) Geneva Switzerland; ^6^ Human Sciences Department University of Rome Guglielmo Marconi Rome Rome Italy; ^7^ Department of Experimental and Clinical Medicine, Section of Psychiatry University of Pisa Pisa Italy; ^8^ Department of Physiology, Anatomy and Genetics, Sir Jules Thorn Sleep and Circadian Neuroscience Institute University of Oxford Oxford UK; ^9^ Centre for Neural Circuits and Behaviour University of Oxford Oxford UK; ^10^ Department of Psychiatry University of Pennsylvania Philadelphia Pennsylvania USA; ^11^ German Center for Mental Health (DZPG) partner site Berlin Berlin Germany

**Keywords:** anxiety, consciousness, depression, dreaming, insomnia, micro‐arousals, rapid eye movement sleep

## Abstract

The world‐wide prevalence of insomnia disorder reaches up to 10% of the adult population. Women are more often afflicted than men, and insomnia disorder is a risk factor for somatic and mental illness, especially depression and anxiety disorders. Persistent hyperarousals at the cognitive, emotional, cortical and/or physiological levels are central to most theories regarding the pathophysiology of insomnia. Of the defining features of insomnia disorder, the discrepancy between minor objective polysomnographic alterations of sleep continuity and substantive subjective impairment in insomnia disorder remains enigmatic. Microstructural alterations, especially in rapid eye movement sleep (“rapid eye movement sleep instability”), might explain this mismatch between subjective and objective findings. As rapid eye movement sleep represents the most highly aroused brain state during sleep, it might be particularly prone to fragmentation in individuals with persistent hyperarousal. In consequence, mentation during rapid eye movement sleep may be toned more as conscious‐like wake experience, reflecting pre‐sleep concerns. It is suggested that this instability of rapid eye movement sleep is involved in the mismatch between subjective and objective measures of sleep in insomnia disorder. Furthermore, as rapid eye movement sleep has been linked in previous works to emotional processing, rapid eye movement sleep instability could play a central role in the close association between insomnia and depressive and anxiety disorders.

## 1 INTRODUCTION

In this review, we aim to highlight the role of rapid eye movement (REM) sleep instability, defined as fragmented or unstable REM sleep (see below), for pathophysiological considerations concerning chronic insomnia (insomnia disorder, ID) and its link to mental illness, especially depression and anxiety disorders. Thus, after providing a short introduction to insomnia, we refer briefly to aetiological/pathophysiological insomnia concepts, sleep perception, insomnia and dreaming, pre‐sleep state and insomnia and, finally, studies directly investigating REM sleep instability, defined by increased numbers of (micro‐)arousals from this sleep state in comparison to good sleepers. We hypothesise that these alterations of REM sleep may constitute a specific phenotype of chronic insomnia, going beyond conceptualisations of insomnia being a sleep disorder mainly characterised by too much wake time during nocturnal sleep. Furthermore, as REM sleep functions have been linked to maintenance of adaptive emotional processes, REM sleep may be a key mechanism linking ID with mental disorders, especially depression and anxiety.

## 2 INTRODUCTION TO INSOMNIA

Approximately 6%–10% of the adult population in industrialised countries suffer from chronic insomnia according to DSM‐IV criteria, defined by difficulties initiating or maintaining sleep with associated daytime symptoms for at least 4 weeks. Women are more frequently afflicted than men, and this difference becomes more pronounced after the age of 45–50 years (Ohayon, 2002). Data on the course of insomnia indicate that once persisting for at least a month, it frequently becomes chronic (Morin et al., 2009). In this chronic form, insomnia is coupled with many unfavourable health outcomes and reduced quality of life (Benz et al., 2023; Kyle et al., 2010). It leads to increased risks for cardiovascular diseases, obesity and the development of diabetes (Anothaisintawee et al., 2016; Benz et al., 2023; Chan et al., 2018; Li et al., 2014), depression (Baglioni et al., 2011; Hertenstein et al., 2019, 2023), anxiety disorders (Hertenstein et al., 2019, 2023), and suicidality/suicide (Pigeon et al., 2012). Insomnia often (25%–60%) accompanies neurological disorders (Mayer et al., 2011), and Yaffe et al. (2014) pointed to evidence that insomnia is involved in the development of cognitive impairment. Insomnia is a risk factor for work disability, sick leave and impaired work performance (Kucharczyk et al., 2012). Not surprisingly, ID is associated with a high burden for the healthcare system and society: 150 billion US dollars have been calculated as direct and indirect costs of insomnia (Reynolds & Ebben, 2017). Data from Germany (DAK Gesundheitsreport; Marschall et al., 2017) show a rise in the prevalence of insomnia from 2009 to 2016, and a parallel increase in hypnotic prescriptions. Besides that, the COVID‐19 pandemic has led to a further significant increase in insomnia prevalence (Morin et al., 2022).

Summarising, chronic insomnia is a highly prevalent disorder, with a strong negative impact on somatic and mental health coupled with high costs for the healthcare system. The DSM‐5 (American Psychiatric Association, 2013) acknowledged this impact by creating the new diagnostic category “Insomnia Disorder” (ID), which was taken up in ICSD‐3 (American Academy of Sleep Medicine, 2014) and ICD‐11 (World Health Organization, 2019), replacing the traditional primary/secondary insomnia dichotomy (Table 1; ICSD‐3 criteria). At the core, ID is defined by sleep complaints coupled with daytime sequelae.

**TABLE 1:** Diagnostic criteria for chronic ID according to ICSD‐3 (American Academy of Sleep Medicine, 2014)

A. The patient reports, or the patient's parent or caregiver observes, one or more of the following:
1. Difficulty initiating sleep.
2. Difficulty maintaining sleep.
3. Waking up earlier than desired.
4. Resistance to going to bed on appropriate schedule.
5. Difficulty sleeping without parent or caregiver intervention.
B. The patient reports, or the patient's parent or caregiver observes, one or more of the following related to the nighttime sleep difficulty:
1. Fatigue/malaise.
2. Attention, concentration or memory impairment.
3. Impaired social, family, occupational or academic performance.
4. Mood disturbance/irritability.
5. Daytime sleepiness.
6. Behavioural problems (e.g. hyperactivity, impulsivity, aggression).
7. Reduced motivation/energy/initiative.
8. Proneness for errors/accidents.
9. Concerns about or dissatisfaction with sleep.
C. The reported sleep/wake complaints cannot be explained purely by inadequate opportunity (i.e. enough time is allotted for sleep) or inadequate circumstances (i.e. the environment is safe, dark, quiet and comfortable) for sleep.
D. The sleep disturbance and associated daytime symptoms occur at least three times per week.
E. The sleep disturbance and associated daytime symptoms have been present for at least 3 months.
F. The sleep/wake difficulty is not better explained by another sleep disorder.

Most recent insomnia guidelines for the USA, Germany and Europe (Qaseem et al., 2016; Riemann, Baglioni, et al., 2017; Riemann, Baum, et al., 2017; Riemann, Espie, et al., 2023) acknowledged the importance and increasing health burden of insomnia, and recommend a thorough differential‐diagnostic procedure followed by cognitive‐behavioural treatment (CBT‐I) as first‐line clinical intervention. It is reasonable to assume that adequate insomnia treatment may not just alleviate the burden of insomnia itself but also have a positive impact on any kind of comorbid disorder (Riemann et al., 2022).

## 3 AETIOLOGICAL AND PATHOPHYSIOLOGICAL ASPECTS OF INSOMNIA

In this review article, we will focus on the hyperarousal concept, however, being fully aware that there are many alternative or competing conceptualisations of insomnia (Espie, 2023; Espie et al., 2006; Harvey, 2002; Kay & Buysse, 2017; Morin, 1993; Perlis et al., 1997; Spielman et al., 1987; Tang et al., 2023). State of the art theories of chronic insomnia emphasise the role of cognitive, emotional and physiological hyperarousal for its development and maintenance (Espie et al., 2006; Harvey, 2002). Own work (Dressle & Riemann, 2023; Morin et al., 2015; Riemann et al., 2010, 2011, 2012, 2015, 2022), and work by others, for example, Bonnet and Arand (2010) and Buysse et al. (2011) summarised that hyperarousal processes play a key role in the pathophysiology of insomnia. Autonomous, neuroendocrine, neuroimmunological, electrophysiological, neuroimaging and psychological studies deliver converging evidence for increased levels of day‐ and nighttime arousal in ID compared with good sleepers. Mirroring the subjective experience of patients with ID having difficulties to “shut down” or to disengage from wakefulness (especially when trying to fall asleep), physiological data reflect increased levels of arousal both during day‐ and nighttime compared with good sleepers, for example, concerning the output of cortisol (Dressle et al., 2022). It is assumed that the permanent hyperarousal in chronic insomnia is triggered by stressful life events and maintained by maladaptive habit formation (e.g. sleep extension and/or irregular sleep scheduling; Spielman et al., 1987), classical conditioning of cortical arousal peri‐sleep onset and during non‐REM (NREM) sleep (Perlis et al., 1997), the incidence of local wakefulness during electroencephalographic (EEG)‐defined sleep (Buysse et al., 2011), and/or an (epi‐)genetic vulnerability to, or the development of the failure to downregulate arousal (Espie, 2002, 2023; Palagini et al., 2014, 2023). A seminal study with fluorodeoxyglucose‐positron emission tomography (FDG‐PET; Nofzinger et al., 2004), which was later replicated in a larger sample (Kay et al., 2016), revealed that patients with ID show smaller declines in relative glucose metabolism from wakefulness to NREM sleep in wake‐promoting regions, including the ascending reticular activating system, the hypothalamus and thalamus, but also in various cortical areas including the insular, cingulate and medial prefrontal cortices.

During NREM sleep, increased cyclic alternating pattern (CAP) rates occur in patients with ID (Chouvarda et al., 2011; Parrino et al., 2009; Terzano et al., 2003). CAP cycles are phasic events consisting of phases with higher (phase A) and lower (phase B) electrophysiological arousal levels thereby mirroring fluctuations of arousal (Terzano et al., 1985; Terzano & Parrino, 2000). The A2 and A3 subtypes of phase A in particular are therefore discussed as markers of unstable, that is, (hyper‐)aroused NREM sleep (Feige et al., 2023).

A potential neurophysiological link between the cortical hyperarousal and polysomnographic (PSG) findings in patients with ID—increased sleep‐onset latency, reduced total sleep time and sleep efficiency, and in particular REM sleep instability (Baglioni, Regen, et al., 2014)—results from the discovery of an essential role for the cerebral cortex in sleep regulation (Krone et al., 2021). Several experiments in mice recently demonstrated that cortical activity fluctuations can modulate REM sleep initiation (Hong et al., 2023), REM episode duration (Wang et al., 2022) and transitions between REM sleep substates (Dong et al., 2022). Especially the prefrontal cortex appears to have a previously unknown function in both preparation and initiation of sleep (Tossell et al., 2023), as well as for the transition into and maintenance of REM sleep (Hong et al., 2023), through direct projections to established sleep‐control centres in the hypothalamus. Together, these animal studies demonstrate that cortical activity changes can lead to rapid vigilance state transitions. Based on this it can be speculated that cortical hyperarousal observed by Nofzinger, Kay and colleagues in human neuroimaging studies might drive subcortical arousal systems and cause (REM) state instability.

Our own summaries (Aquino et al., 2024; Riemann et al., 2015) of neuroimaging studies concluded that corticolimbic overactivity (during day‐ and nighttime) interferes with sleep‐promoting neural processes in ID. We further suggested that REM sleep may be even more prone to disturbances in patients with insomnia as evidenced by the relationship of this sleep state to increased subjective wake time and increased frequency of micro‐arousals compared with NREM sleep (Feige et al., 2008, 2013, 2023; Riemann et al., 2012; Riemann, Dressle, et al., 2023). Pre‐sleep arousal (measured on a psychometric level) is also specifically enhanced in insomnia (Dressle et al., 2023; Spiegelhalder et al., 2012; Vochem et al., 2019), and this is paralleled by electrophysiological activity in the awake resting state (Colombo et al., 2016; Feige et al., 2017), where patients with insomnia compared with good sleepers displayed more beta and less alpha power during an eyes open/eyes closed paradigm directly prior to sleep.

When considering classical PSG, a meta‐analysis (Baglioni, Regen, et al., 2014) demonstrated significant disturbances of sleep continuity (prolonged sleep latencies, more nocturnal wake time) and less total sleep time in ID compared with good sleepers. It is noteworthy that these changes, although statistically significant, were not of a large magnitude: total sleep time, for example, was shortened by an average of 25 min, whereas on a subjective level, this difference amounted up to 120 min. For sleep architecture, this meta‐analysis found reduced amounts of slow‐wave sleep and REM sleep in ID. The reduction in REM sleep was thought to be related to the already mentioned fragmentation or instability of REM sleep described in samples of patients with ID. With respect to the spectral analysis of sleep, it has been shown more or less consistently that sleep in ID is characterised by increased fast frequencies in the sigma, beta and gamma range (for an overview, see Feige et al., 2013).

Concerning REM sleep regulatory mechanisms and functions, the reader is referred to Casaglia and Luppi (2022) and other articles in this special issue of the *Journal of Sleep Research* devoted to REM sleep. In brief, the neuroanatomy, neurochemistry and neurophysiology of REM sleep is uniquely different from the wake state and all other NREM sleep states. Hobson and McCarley (Hobson et al., 1975; McCarley, 1982) were the first to suggest a reciprocal interaction model of NREM and REM sleep regulation, governed by cholinergic (REM on) and aminergic (REM off) neurons mainly located in the brainstem. The aminergic branches were thought to consist mainly of the dorsal raphe nuclei and the locus coeruleus (LC), areas that were assumed to be more or less silent during REM sleep. More recent work (Kjaerby et al., 2022; Van Someren, 2021) expanded this view towards the activity of the LC during sleep with sophisticated experimental approaches. It was shown that norepinephrinergic (NE) output of the LC during NREM sleep drives micro‐arousal activity and is involved in processes of memory consolidation. In contrast to healthy sleep, unstable or restless REM sleep signals that the LC is not silent in patients with insomnia during the REM sleep state, probably negatively impacting synaptic brain plasticity (Van Someren, 2021). It is speculated that compounds blocking or downregulating NE during sleep might be of benefit for clinical conditions related to hyperarousal‐like post‐traumatic stress disorder (PTSD), borderline personality disorder or insomnia (Broese et al., 2012).

## 4 PERCEPTION OF SLEEP

An enigma that still needs to be deciphered is the marked discrepancy between the subjective experience of sleep (usually measured by sleep questionnaires) and objective, that is, PSG findings in ID. It is a hallmark of “healthy” sleep that only rarely, even if wake phases and active behaviour were clearly present in the PSG, any memory exists for the time interval between going to sleep and waking up. This normative phenomenon is sometimes referred to as the mesograde amnesia of sleep (Perlis et al., 1997, 2001; Wyatt et al., 1994, 1997). In contrast, insomnia is characterised by the subjective report of nocturnal wake phases that are perceived to be much longer than determined by PSG (for an overview, see Harvey & Tang, 2012). This discrepancy led to the terms “pseudo‐insomnia”, “sleep state misperception” or “paradoxical insomnia” for patients with a “relatively” normal sleep continuity and architecture, in spite of massive subjective complaints (Edinger & Krystal, 2003). The discrepancy between subjective/objective data might be interpreted as reflecting a specific phenotype of insomnia; however, alternatively, this discrepancy might be distributed on a continuum. Furthermore, we are not aware of any data elucidating how stable this discrepancy is over several nights or weeks and how it responds to different therapeutics. Harvey and Tang (2012) listed 13 theoretical approaches to explain this difference—our own hypothesis focusses on REM sleep “instability” as the underlying mechanism for this discrepancy (see below). An alternative, though not contradictory, explanation is offered by the concept of local sleep/local wakefulness. It has been shown that sleep may not be such a homogenous phenomenon as previously assumed. Slow waves are not uniformly distributed across the cortex (Nir et al., 2011), but can occur asynchronously across different areas of the brain (Huber et al., 2004) and locally even during behavioural wakefulness for short periods (Vyazovskiy et al., 2011). Siclari and Tononi (2017) suggest that “the coexistence of local sleep‐like and wake‐like patterns in different brain areas is characteristic of different sleep disorders” (Siclari & Tononi, 2017, p. 1). This same concept might be applicable to ID, except in this instance local wakefulness would occur during what is otherwise sleep. This concept was first proposed by Buysse and colleagues (Buysse et al., 2011), and it represents a natural complement to the aberrant forms of wakefulness proposed by both the neurocognitive model and REM instability models. In all three instances, chronic insomnia is not only characterised by increased nocturnal wakefulness (as assessed with EEG/PSG), but also has hybrid forms of NREM and REM sleep that are difficult for the individual to perceive as sleep. Stephan and Siclari (2023) provocatively stated: “Reconsidering sleep perception in insomnia: from misperception to mismeasurement”, “stressing that our so‐called” objective measurement methods of insomnia (e.g. PSG) might be to blame for this discrepancy, but not the patients with ID. Thus, the authors suggests that a shift towards increased cortical activation throughout the sleep state is associated with the experience of feeling awake.

### 4.1 REM sleep instability

A well‐replicated finding in patients with ID is an increase in micro‐arousals during sleep (Van Someren, 2021). Regarding this, an important role for a disturbance of REM sleep (either termed REM sleep instability or restless REM sleep) has been postulated (Feige et al., 2008, 2023; Riemann et al., 2012; Riemann, Dressle, et al., 2023) regarding the pathophysiology of insomnia and, in particular, for the altered perception of sleep and the inability to downregulate hyperarousal. According to the current version of the American Academy of Sleep Medicine Manual for the Scoring of Sleep and Associated Events (Berry et al., 2020), arousals are basically defined as shifts in EEG frequency towards theta, alpha and/or frequencies exceeding 16 Hz, which must occur for at least 3 s. During REM sleep, these abrupt EEG frequency shifts must also be accompanied by an increase in muscle tone.

**FIGURE 1:**
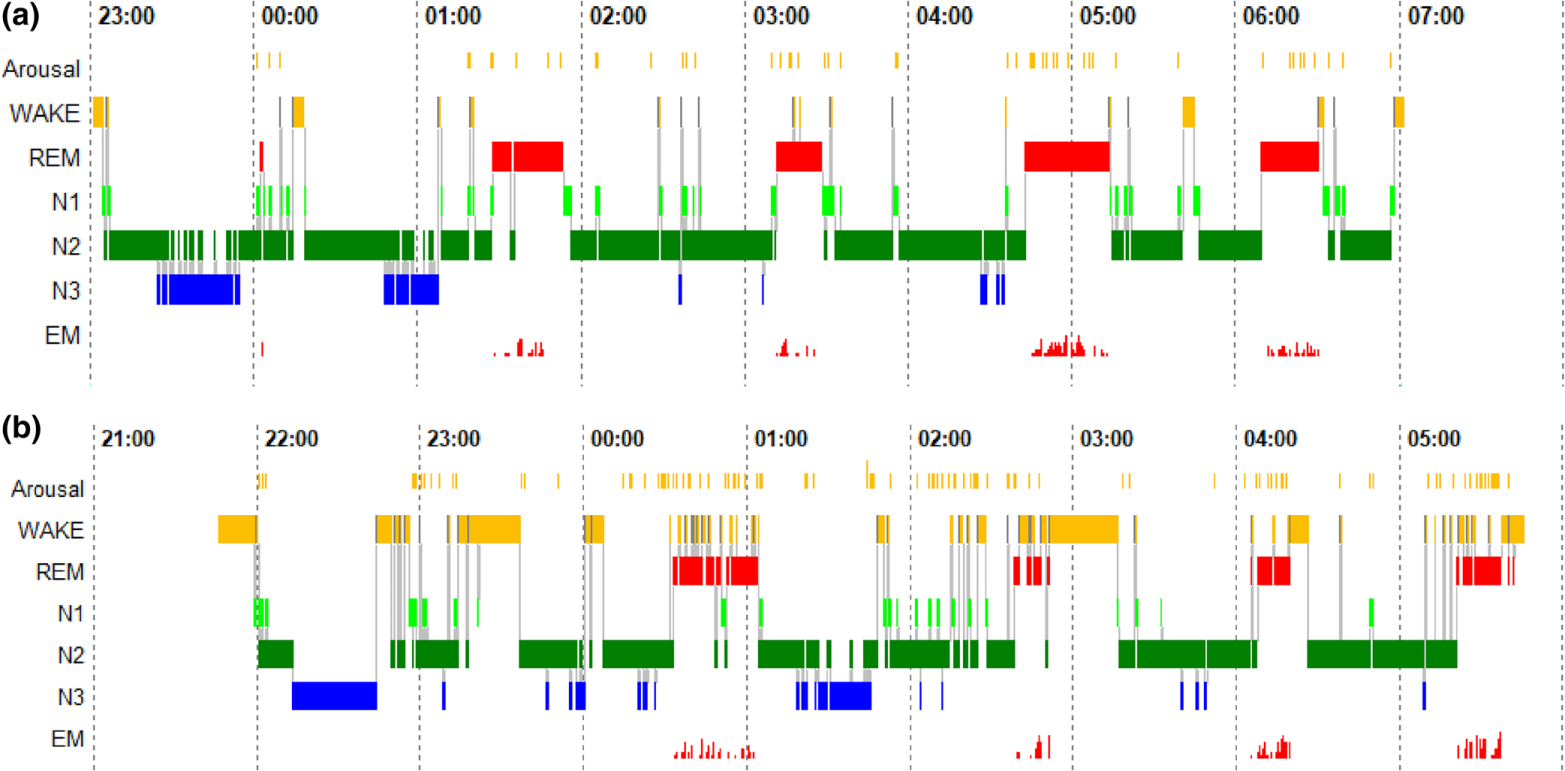
Polysomnographic (PSG) profiles of a good sleeper (upper panel; a) and a patient with insomnia (lower panel; b). The *y*‐axis displays arousal (micro‐arousals), wake and sleep stages (rapid eye movement [REM], stage N1, N2 and N3), as well as eye movements (EM). The *x*‐axis is the time axis.

Figure 1 highlights the sleep patterns of ID compared with good sleepers. Figure 1 gives full‐night data comparing second nights of a good sleeper (age‐ and gender‐matched, approximately 50 years old) with a patient with ID in the sleep laboratory.

For the first time, this was highlighted by the finding that in patients with ID, REM sleep duration was related to subjectively reported wake time, and the increase in micro‐arousals was most pronounced during REM sleep (Feige et al., 2008). In this study, 100 good sleeper controls and 100 patients with ID were investigated for 2 nights in a drug‐free state with routine PSG in the sleep laboratory. The analysis of night 2 not only looked at routine parameters of sleep continuity, sleep architecture and REM sleep, but encompassed scoring of micro‐arousals during all stages of sleep. REM sleep time was found to contribute to subjective wake time, which, together with the REM time reduction relative to good sleepers, was interpreted as a sign of a qualitatively modified REM sleep. Interestingly, the strongest difference between both groups was found for REM‐sleep‐associated micro‐arousals, with a significant increase in ID compared with good sleepers. To obtain more direct evidence for the involvement of REM sleep, a next study (Benz et al., 2020; Feige et al., 2018) with 4 nights in the laboratory investigated NREM (stage N2) and REM sleep awakenings in nights 3 and 4 in subgroups of 27 good sleepers and 27 patients with ID. All subjects were awoken three times out of N2 and three times out of REM sleep (not including the first sleep cycle) either in night 3 or night 4 according to a counterbalanced design. The main outcome parameter was the judgement of the acute state when awoken, that is, sleeping versus being awake. When awakened from REM sleep, patients with ID reported being awake more frequently than good sleepers did (Feige et al., 2018), but not when awakened from NREM sleep. It was found that REM sleep spectral power was related to this increased perceived wakefulness (in particular, lower delta, theta and alpha power in the minute prior to the REM awakenings; Benz et al., 2020). Additionally, mood ratings performed after the awakenings revealed a progressive worsening of mood in patients with ID after REM sleep awakenings, which did not occur in stage N2 awakenings and in good sleepers. In a next step, Feige et al. (2021) looked at the continuous measurement of auditory event‐related potentials (ERPs; more than 10,000 stimuli per night) over the course of the night in 50 good sleepers and 50 patients with ID. In contrast to good sleepers, patients with ID exhibited reduced P2 amplitudes in phasic REM sleep exclusively, but not in any other sleep state. Further topographical analysis showed that this was most likely due to increased mismatch negativity in ID, mirroring increased automated change detection in the auditory system and a concomitant orienting response. The interpretation given was that individuals with ID are, especially during phasic REM sleep, prone to sensory afference, thus contributing to the perception of being awake instead of being asleep or dreaming. These series of experiments emphasise the important role of REM sleep and its instability in insomnia. In an ongoing study, we are now investigating the impact of two bedtime stories (confrontation: a story about insomnia and its consequences; versus distraction: a very funny story without any insomnia‐related content) on PSG‐recorded sleep and ERPs in ID.

Wassing et al. (2016), Wassing, Benjamins, et al. (2019), and Wassing, Lakbila‐Kamal, et al. (2019) conducted experiments to further elucidate the underlying mechanisms, and showed that REM sleep disturbances hamper the nocturnal adaptation of limbic circuits (as a response to shameful experiences). As a consequence, difficulties in resolving distress may occur, which may be a crucial component of the development and maintenance of hyperarousal as well as the risk to develop other mental disorders. Interestingly, unperturbed REM sleep is accompanied by the prolonged silence of the LC, which in turn is coupled to a decrease of cerebral NE (Kjaerby et al., 2022). REM sleep is supposed to serve the limbic reactivation of emotional memory traces. Van Someren (2021) postulated that increased levels of NE during restless REM sleep could disrupt synaptic plasticity processes underlying the adaptation of neuronal engrams that represent distress, and possibly even result in further sensitisation. It has also been suggested that low NE during REM sleep is central to the restoration of NE tone, and to allow for low tonic and high phasic LC activity during wakefulness (Kjaerby et al., 2022). In the same publication, it is also hypothesised that undisturbed REM sleep over the course of the night, because of the silence of the LC, allows for the integration of emotional memories and helps to balance the emotional equilibrium of the individual.

Given these theoretical considerations backed by experimental data, REM sleep perturbations in ID might also be interpreted as the underlying physiological correlate of emotional alterations found in ID (Meneo et al., 2023) and a major pathway to develop significant psychopathology during the chronic course of the disorder.

## 5 PRE‐SLEEP COGNITIVE AROUSAL, NOCTURNAL MENTATION AND EMOTION REGULATION

### 5.1 The relationship between cognitive processes during wakefulness and those during sleep in patients with ID

When discussing cognitive processes in patients with ID, at least two types of cognitions and their interaction must be distinguished. These are, first, dysfunctional cognitive processes during wakefulness and, second, those that occur during sleep, that is, dreams. At present, researchers in the field, rather than referring to “dreaming”, prefer to study “sleep mentation/cognition” as a dependent variable. This is defined as any description of experiences remembered upon awakening that occurred during previous sleep. This serves to avoid bias and preconceptions related to what a dream should be. In fact, it is now widely acknowledged that any kind of cognitive process/mentation can occur in sleep stages N1, N2, N3 or REM sleep (Siclari et al., 2013). However, 77% of awakenings from REM sleep and 34% of awakenings from NREM sleep lead to recall with content. It is assumed that from wake to sleep, especially in stages N1 and N2, more thought‐like mentation may occur. N3 is likely to be relatively “silent” with respect to sleep mentation (Siclari et al., 2013), whereas the REM sleep state offers the chance or sets the stage for vivid visual‐hallucinatory experiences, sometimes coupled with high levels of emotionality (Siclari et al., 2013). Overall, there generally appears to be a continuum of cognitive processes during sleep, oscillating between more thought‐like experiences and the classical vivid dream experience. As will be discussed below, a central pathological mechanism of ID may involve a disruption of these complex, finely balanced nocturnal cognitive processes. This disruption is likely to be closely linked to dysfunctional cognitions during wakefulness, particularly in the period just before sleep.

In her cognitive model of insomnia, Harvey (2002) and (2005) posits that patients with ID demonstrate increased worry about disturbed sleep and its daytime consequences, resulting in emotional distress and signs of physiological arousal and anxiety. This cognitive arousal would then increase selective attention towards sleep‐related threats, which would increase the likelihood of detecting cues that become a subject of even more worry, thereby forming a vicious circle that maintains high levels of cognitive arousal. Indeed, pronounced worry and rumination is a consistent finding in insomnia research occurring both in the pre‐sleep period (Dressle et al., 2023; Hertenstein et al., 2015; Kalmbach et al., 2020; Nicassio et al., 1985; Palagini et al., 2017; Vochem et al., 2019) and during the day (Kallestad et al., 2010; Palagini et al., 2015). But how could this pre‐sleep state cognitive activation influence sleep, especially REM sleep? The widely acknowledged continuity hypothesis of dreaming (Schredl, 2003) assumes that sleep mentation reflects some form of continuity with experiences during the day. In line with this, Schredl (2009) summarised (based on a relatively scarce literature basis), that patients with ID experience themselves more negatively in their dream‐life and that their acute wake‐life concerns were reflected in dream narratives. Thus, it seems justified to postulate that one major concern of people with ID, namely the perceived inability to sleep properly, which provokes pre‐sleep arousal, may be dominantly reflected in their subsequent dream experiences. Interpreting their results on sleep mentation from repetitive awakenings from NREM and REM sleep in patients with ID, Feige et al. (2018) hypothesised that this continuation of the content and emotional tone of pre‐sleep cognitions in patients with ID could subsequently result in a disturbed capacity to subjectively discriminate between wakefulness and sleep, thereby contributing to the phenomenon of sleep misperception in patients with ID. Apart from an altered rate of state judgements especially for REM sleep (judging REM awakenings more frequently as having been awake), mood ratings over succeeding REM periods indicated an increase in negative mood over the course of the night in sleepers with ID, but not in good sleepers, in this study. These results interestingly resemble earlier work in subjects with depression where we were able to show that mood ratings over the course of successive REM sleep awakenings during the night, compared with good sleepers, reflected increased amounts of negative mood towards the morning hours (Riemann et al., 1990).

### 5.2 The role of dysfunctional cognitive coping strategies

In addition to the continuity of cognitive content between the pre‐sleep period and sleep, there is likely an additional detrimental influence of dysfunctional strategies applied by patients to overcome excessive cognitive arousal in the pre‐sleep period. For example, it is quite clear that most patients afflicted with insomnia try to suppress unwanted thought processes and associated features of physiological arousal in the pre‐sleep period, however, frequently to no avail (Harvey, 2001, 2002, 2005). It was demonstrated that by asking healthy sleepers to suppress unwanted thoughts prior to sleep, dream content over the next week was characterised by an even increased frequency of the suppressed content (Kröner‐Borowik et al., 2013). These findings have been confirmed by other authors (Bryant et al., 2011; Malinowski, 2017; Malinowski et al., 2019; Taylor & Bryant, 2007; Wegner et al., 2004). Therefore, it could be speculated that patients with ID, by trying not to think about their insomnia prior to sleep, in the end perpetuate the experience of endless ruminations, racing thoughts and the experience of being awake during their sleep. Thought suppression as an emotion regulation strategy would also be coupled with increased physiological arousal, mimicking the situation of insomnia. Interesting, recent findings from our group, obtained through an ecological momentary assessment design, found increased self‐report of use of suppression/maladaptive emotion regulation strategies in individuals with ID compared with good sleepers (Baglioni et al., 2023). This might exert its influence primarily during REM sleep and thus interfere with its postulated function of “emotional memory processing” (Walker & van der Helm, 2009).

Another dysfunctional strategy applies to the modality of cognitive processes. In general, worry and rumination can appear in a more verbal or in a visual, imagery form (Nelson & Harvey, 2002, 2003). Mental imagery is thought to be more likely than verbal thought processes to trigger aversive emotions (e.g. fear and anxiety) and associated physiological arousal (Davey & Wells, 2006; Stöber et al., 2000; Toh & Vasey, 2017). In addition, Nelson and Harvey (2003) found that patients with ID rate their imagery in the pre‐sleep period as more distressing than good sleepers. As a result, they may undergo “imagery control”, that is, the shift of cognitive processing from an image‐dominated mode to a more verbal mode (Harvey, 2002; Schmidt et al., 2011). In line with this assumption, Nelson and Harvey (2003) found that mental imagery in the pre‐sleep period is not only more negatively valenced in patients with ID, but also occurs less frequently overall compared with control subjects with good sleep. However, this strategy is likely highly dysfunctional. Most importantly, applying this strategy adversely affects normal changes in cognitive processing during the pre‐sleep period where an increase in visual and hallucinatory characteristics is typically observed (Lemyre et al., 2020). Given the assumed continuity between waking and nighttime cognitive activity, it is likely that the following sleep mentation is also verbally dominated, rather than being “dream‐like” (i.e. visual, hallucinatory, bizarre). In this way, nighttime cognitive activity may become even more difficult to distinguish from the normal daytime cognitive activity of thinking and ruminating, thereby negatively affecting perceived sleep depth (Stephan et al., 2021) and fostering sleep misperception. These factors, in turn, contribute to heightened insomnia‐related worries. An increase in micro‐arousals during REM sleep could directly foster this effect by paving the way for enhanced recall of nocturnal cognitions.

Keeping in mind associations between (micro‐)arousals during sleep and the activity of the LC (Kjaerby et al., 2022; Van Someren, 2021), an overactive LC either during NREM or especially during REM sleep might be at the bottom of increased cognitions and their recall in chronic insomnia.

### 5.3 Cognitive arousal and emotion regulation

The concepts of cognitive and emotional arousal are strongly interrelated. Cognitive arousal frequently results in emotional distress, and the relationship between cognitive and somatic signs of hyperarousal is assumed to be mediated by emotional reactivity (Baglioni, Spiegelhalder, et al., 2010; Harvey, 2002). In this regard, emotional arousal is not only a byproduct of distressing cognitions, but emotion regulation might be dysfunctional in patients with ID, as has been suggested by several authors (Baglioni, Spiegelhalder, et al., 2010; Meneo et al., 2023). This line of research studied correlations between daily mood states and sleep quality, emotional reactivity and sleep, or utilised paradigms of mood induction (Baglioni, Spiegelhalder, et al., 2014) or ecological momentary assessment of mood to describe relationships between affective states/mood regulation and sleep quality in good sleepers and chronic ID (Baglioni et al., 2023). Summarising, days with more negative emotions tend to be followed by nights with more sleep alterations, whereas longer wake episodes at night were followed by increased variations in emotions during the day. Concerning the interaction between sleep quality and emotional valence, impaired sleep quality tends to correlate with high negative and low positive emotions. The importance of these findings to understand relationships between insomnia and mental illness, especially anxiety and depression, will be further highlighted below.

## 6 CHRONIC INSOMNIA, REM SLEEP INSTABILITY AND DYSFUNCTIONAL EMOTION REGULATION: A MAJOR PATHWAY TO DEVELOP ANXIETY AND DEPRESSION?

Insomnia disorder and other psychopathological conditions like affective and anxiety disorders show large overlap, both in terms of relevant symptoms and proposed pathophysiological models. For example, besides being a key model in research on ID, the concept of hyperarousal rooting in the cortisol, noradrenergic LC and orexin systems has also been proposed in psychophysiological models of anxiety and depression (Geiger et al., 2014; Gottschalk et al., 2019; Hoehn‐Saric & McLeod, 2000; Xie et al., 2024) as well as its treatment (Brooks et al., 2019; Neufang et al., 2019). Also, impairments of sleep continuity occur in most mental disorders (Baglioni et al., 2016), particularly so in depression, anxiety disorders and PTSD (Richards et al., 2020). Thus, insomnia qualifies as a transdiagnostic factor for psychopathology in general, and especially for mood and anxiety disorders. Regarding REM sleep, ID is characterised by an increase in micro‐arousals and an overall decrease in REM sleep duration. Interestingly, the typical pattern of REM sleep disturbance in depressive disorders includes a decrease in REM latency, a prolongation of the first REM period and, probably most importantly, an increased REM density (Baglioni et al., 2016; Riemann et al., 2001, 2020). Early reports on REM sleep interpreted this pattern of results as reflecting an overall increased REM pressure as a result of prolonged REM deprivation (Snyder, 1972). Subsequently, the finding of insomnia‐specific REM sleep disturbances together with the close association between insomnia and depression gave rise to the assumption that preceding periods of insomnia are at least partly responsible for (periods of) disinhibited REM sleep in depression (Riemann et al., 2012, 2020). An increase in REM pressure has also been found in PTSD, but not in other anxiety disorders such as panic disorder (Baglioni et al., 2016).

Given this large overlap, it seems reasonable to assume that the relations between insomnia and depression/anxiety are bi‐directional. Indeed, insomnia has been shown to be a significant predictor of depressive and anxiety disorders (Baglioni et al., 2011; Hertenstein et al., 2019, 2023). Insomnia treatment was even shown to be able to prevent mood and anxiety disorders (Christensen et al., 2016). Nevertheless, it is still difficult to understand how ID is related to psychopathology on a mechanistic level. A promising approach focuses on the role of REM sleep for emotion regulation. The described studies by Wassing and colleagues (Wassing et al., 2016; Wassing, Benjamins, et al., 2019; Wassing, Lakbila‐Kamal, et al., 2019) show that a fragmented REM sleep may disturb the nocturnal adaptation of limbic circuits, leading to difficulties in resolving distress. Undisturbed REM sleep is accompanied by the prolonged silence of the LC, which in turn is coupled to a decrease of cerebral NE (Kjaerby et al., 2022). This allows for the integration of emotional memories and helps to balance the emotional equilibrium of the individual. Increased levels of NE during restless REM sleep, in turn, could disrupt synaptic plasticity processes underlying the adaptation of neuronal engrams that represent distress, and possibly even result in further sensitisation (Van Someren, 2021). In the long term, this could lead to an accumulation of arousal and distress, paving the way for (comorbid) depressive and anxiety disorders (Van Someren, 2021).

**FIGURE 2:**
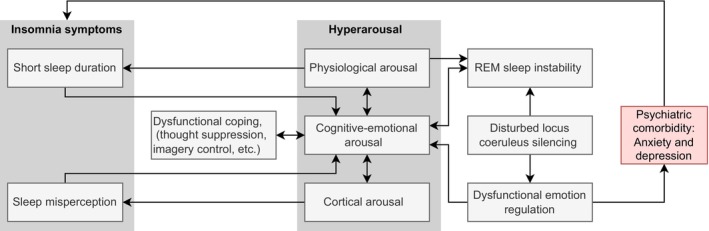
Comprehensive model of chronic insomnia, rapid eye movement (REM) sleep instability, hyperarousal, dysfunctional emotion regulation, and anxiety and depression.

Indirect support for the role of potentially REM sleep‐related disturbances in emotion regulation can be derived from experimental studies that have demonstrated that patients with insomnia compared with those with good sleep report more negative emotions (McCrae et al., 2008; Scott & Judge, 2006). Psychophysiological studies have also demonstrated that patients with insomnia show an emotional bias to sleep‐related stimuli with negative valence (Baglioni, Lombardo, et al., 2010; Baglioni, Spiegelhalder, et al., 2014). Overall, these data are consistent with the perspective that sleep is a fundamental psychophysiological process owning a key role in the regulation of stress and emotion (Hagger, 2010; Palmer & Alfano, 2017). In several overview papers (Palagini et al., 2014; Riemann et al., 2001, 2020, 2022), we have outlined in more detail the relationships especially between sleep, insomnia, depressive and anxiety disorders. Figure 2 summarises current thinking about chronic insomnia, REM sleep instability hyperarousal, dysfunctional emotion regulation, and anxiety and depression.

## 7 FUTURE PERSPECTIVES

Where do we go from here? First of all, we need to further characterise REM sleep instability in larger samples of patients with ID, and check for age‐ and sex differences. Can the relationship between REM sleep instability and the subjective–objective discrepancy be confirmed? Does this constitute a distinct phenotype of ID or is it a continuous variable? Is REM sleep instability specific for ID or does it occur also in ID comorbid with other conditions like anxiety or depression? What about the reliability of REM sleep instability and the subjective–objective discrepancy? Repetitive measurement approaches are needed to delineate how stable these phenomena occur in ID. In addition, causal assumptions about the relationship between REM sleep instability in patients with ID and depression/anxiety are limited by the fact that they are derived only from theoretical considerations and cross‐sectional studies. Future studies that investigate the course of disease in patients with ID longitudinally, repeatedly examining sleep, are needed. Another direction for future research pertains to the need for a deeper understanding of the influences of various therapeutics (pharmacological versus cognitive‐behavioural) on REM sleep instability and the subjective–objective discrepancy. Maurer et al. (2024) in a secondary analysis of a trial of sleep restriction (SR; probably the most powerful CBT‐I intervention) observed positive effects on an index of REM sleep fragmentation after 1 week of SR therapy. Nevertheless, no significant effect was observed at week 4 (post‐treatment). Therefore, it should be considered how treatments could be improved to achieve more specific and longer‐lasting effects on REM sleep instability. In this context, neuroscientific studies in animals and humans are needed to better understand causal relationships between cortical and systemic hyperarousal, activity of the LC and the NE system, the cortisol and orexin systems, REM sleep instability, ID, emotional regulation and the development of anxiety and depressive disorders. It would also be interesting to see to what extent these variables might have differential‐therapeutic value when choosing between treatments. Furthermore, what about treatments specifically targeting the NE or the orexin system—are they superior to classical hypnotics?

While new CBT approaches suggest that psychotherapy should focus more on transdiagnostic processes (Hayes & Hofmann, 2018), sleep continuity (versus discontinuity, or insomnia) still received little attention in clinical practice both from CBT and psychiatric clinical protocols. Sleep is a vital health issue and, given the fact that sleep alterations are commonly observed in patients with psychopathology, it is reasonable to suggest that basic sleep CBT modules should be considered standardly in mental healthcare.

In addition, the role of REM sleep instability in other mental disorders needs to be better characterised. For example, in PTSD, a mental disorder characterised by marked hyperarousal and symptoms of insomnia, alterations in REM sleep are also described. These include a decrease in the proportion of REM sleep and REM sleep fragmentation (Insana et al., 2012; Zhang et al., 2019), the latter of which being potentially associated with the development of PTSD following acute traumatisation (Mellman et al., 2002; Murkar & De Koninck, 2018). From this perspective, we need a deeper understanding of which aspects of REM sleep disturbance are disorder‐specific and which might even qualify as transdiagnostic process that occurs in many mental disorders that involve disturbances in emotional processing.

## AUTHOR CONTRIBUTIONS

**Dieter Riemann:** Conceptualization; writing – original draft; writing – review and editing; project administration; supervision; methodology; visualization. **Raphael J. Dressle:** Conceptualization; writing – original draft; writing – review and editing; project administration; visualization. **Fee Benz:** Conceptualization; writing – review and editing. **Kai Spiegelhalder:** Conceptualization; writing – review and editing. **Anna F. Johann:** Conceptualization; writing – review and editing. **Christoph Nissen:** Conceptualization; writing – review and editing. **Elisabeth Hertenstein:** Conceptualization; writing – review and editing. **Chiara Baglioni:** Conceptualization; writing – review and editing. **Laura Palagini:** Conceptualization; writing – review and editing. **Lukas Krone:** Conceptualization; writing – review and editing. **Michael L. Perlis:** Conceptualization; writing – review and editing. **Katharina Domschke:** Conceptualization; writing – review and editing. **Mathias Berger:** Conceptualization; writing – review and editing. **Bernd Feige:** Conceptualization; writing – original draft; writing – review and editing; supervision; visualization.

## CONFLICT OF INTEREST STATEMENT

The authors declare no conflicts of interest.

## Data Availability

Data sharing is not applicable to this article as no new data were created or analyzed in this study.
